# Effects of Notch Filters in Correcting Color Vision Deficiency: A Quantitative Clinical Trial

**DOI:** 10.3390/diagnostics16091347

**Published:** 2026-04-29

**Authors:** Jin-Cherng Hsu, Chia-Ying Tsai, Tzu-Ning Cheng, Chien-Chang Yen, Hsing-Yu Wu, Yung-Shin Sun

**Affiliations:** 1 Department of Physics, Fu Jen Catholic University, New Taipei City 242062, Taiwan; 054326@mail.fju.edu.tw (J.-C.H.); nel2989@gmail.com (T.-N.C.); 2 Center for Astronautical Physics and Engineering, National Central University, Taoyuan 237209, Taiwan; 3 Department of Medicine, College of Medicine, Fu Jen Catholic University, New Taipei City 242062, Taiwan; 140230@mail.fju.edu.tw; 4 Department of Ophthalmology, Fu Jen Catholic University Hospital, New Taipei City 242062, Taiwan; 5 Graduate Institute of Biomedical and Pharmaceutical Science, Fu Jen Catholic University, New Taipei City 242062, Taiwan; 6 Department of Mathematics, Fu Jen Catholic University, New Taipei City 242062, Taiwan; ccyen@mail.fju.edu.tw; 7 Taiwan Space Agency, Hsinchu City 300091, Taiwan; 8 Department of Optics and Photonics, National Central University, Taoyuan 237209, Taiwan

**Keywords:** color vision deficiency (CVD), color blind glasses, notch filter, Farnsworth–Munsell 100 Hue test, D-15 panel test, total error score (TES), confusion angle, confusion index (C-index)

## Abstract

**Background/Objectives:** Color vision deficiency (CVD) arises from the absence or dysfunction of one or more cone photoreceptors in the retina, resulting in impaired color discrimination. Although inherited CVD cannot be cured, optical compensation strategies such as color-filtering glasses have been developed to enhance color perception. However, quantitative clinical evaluations of their corrective efficacy remain limited. This study aimed to assess the effectiveness of notch filter-based color blind glasses in improving color perception and discrimination in individuals with CVD. **Methods:** Notch filters were employed as color correction lenses, and clinical assessments were conducted to evaluate their impact on human color perception. Subjects underwent standardized color vision tests, including the Color Bridge test, Farnsworth-Munsell 100 Hue test, and D-15 panel test, both before and after wearing the glasses. Outcomes were quantitatively analyzed using total error score (TES), confusion angle, and confusion index (C-index) to determine changes in color discrimination performance. **Results:** Quantitative analysis demonstrated that wearing the notch filter glasses amplified color differences along confusion lines. In clinical trials, 83% of subjects showed improved color discrimination in the F-M 100 Hue test, with TES reductions between 6.67% and 50.00%. Furthermore, D-15 panel testing revealed that 67% of participants exhibited a decreased C-index and reduced scatter index (S-index), with specific cases shifting from deficient to normal color perception (C-index < 1.6). These results indicate that the filters effectively mitigate symptoms of color vision deficiency by increasing perceptual contrast. **Conclusions:** Notch filter-based color correction glasses can enhance chromatic discrimination in individuals with CVD by increasing perceptual color contrast. These findings provide practical insights for the optimization and fabrication of color vision correction eyewear utilizing spectral notch filtering strategies.

## 1. Introduction

Color vision deficiency (CVD) is a general term for disorders characterized by abnormal color perception. The condition was first described by British chemist John Dalton, who published a scientific paper in 1798 detailing his own atypical color vision [1]. Physiologically, human color perception originates from the stimulation of three types of cone cells in the retina by incident light. These cone cells respond to different wavelength ranges and are categorized based on their spectral sensitivities into short (S), medium (M), and long (L) types [2]. The L cones are most sensitive to wavelengths between 535 and 575 nm, the M cones to 500–550 nm, and the S cones to 400–450 nm. Although L, M, and S cones are commonly referred to as red, green, and blue receptors in informal terms, they do not correspond precisely to specific colors. Color perception arises from the differential responses of these cones, which are subsequently processed by the visual cortex and other related brain regions. The LMS color space can be constructed by measuring the spectral sensitivities of the three cone types [3].

CVD can be either congenital (genetically inherited) or acquired. Acquired CVDs can vary in severity, may affect one eye more than the other, and often indicate underlying health conditions. They may result from ocular diseases, neurological disorders, certain medications, or toxic exposures, aging, or nutritional deficiencies [4,5,6,7,8]. In some cases, such deficiencies can be reversed with medical treatment [9,10]. Congenital CVD, caused by genetic factors, may or may not be partially correctable using color vision correction glasses or other visual aids, depending on the type. However, current medical technology cannot cure congenital CVD. Congenital cases are typically categorized into three main types: monochromacy, dichromacy, and anomalous trichromacy. Monochromacy includes rod monochromacy and cone monochromacy, in which only one type of photoreceptor functions normally while the others are nonfunctional [11,12]. Individuals with monochromacy can perceive visual information only in grayscale (black, white, and shades of gray). This type of CVD cannot be corrected with color vision correction glasses. Dichromats lack one type of cone cell and therefore perceive colors using combinations of the remaining two cone types. This condition is further classified into protanopia (absence of L cones), deuteranopia (absence of M cones), and tritanopia (absence of S cones) [13]. Protanopia and deuteranopia are often grouped as red-green CVD, characterized by difficulty distinguishing between red and green. Anomalous trichromacy, often referred to as color weakness, is characterized not by the absence of cone types but by functional abnormalities in one or two types of cones [14]. This condition can often be corrected or improved using correction glasses. Anomalous trichromacy is further classified based on the affected cone type as protanomaly (abnormal L-cone function), deuteranomaly (abnormal M-cone function), and tritanomaly (abnormal S-cone function) [15]. These abnormalities may involve either reduced sensitivity or a spectral shift in peak sensitivity. As reported, approximately 8% of men and 0.5% of women are affected by various forms of CVD, with red-green being the most common type [16]. Therefore, this study investigates the use of notch filter-based glasses for the correction of red-green CVD.

Currently, a variety of commercial products for CVD correction are available, including tinted glasses, lenses, optoelectronic glasses, mobile applications, digital tools, and even experimental gene therapies [17,18]. Tinted glasses and lenses typically work by filtering specific wavelengths of light to enhance color contrast, making it easier for users to distinguish between confusing color pairs [19]. One of the most widely known brands is EnChroma, which produces glasses that use multi-band notch filter technology [20,21]. These lenses selectively reduce overlapping wavelengths between the red and green cones in the eye, thereby enhancing the perceived separation between these colors. EnChroma glasses are especially effective for individuals with protanomaly or deuteranomaly. However, their effectiveness can vary based on lighting conditions and the user’s specific type of color deficiency. Another major brand is Pilestone, which offers a broader range of lens types, each optimized for different kinds of CVD and for different settings [22]. Pilestone lenses are often more affordable and come with options for both corrective and non-corrective prescriptions. In addition to glasses, researchers and companies have explored tinted contact lenses, such as those developed by X-Chrom and newer experimental designs that use dyed sections or embedded nanoparticles to alter spectral transmission [23]. Moreover, several mobile apps and software solutions support colorblind users by simulating normal vision, labeling colors in real time, or altering screen colors to improve contrast [24,25]. More recently, gene therapy offers a promising approach to correcting inherited CVD, particularly red-green deficiencies caused by mutations in the opsin genes [26,27]. While no human clinical trials have been approved yet, ongoing research continues to explore the safety, efficacy, and long-term outcomes of gene therapy as a potential cure for CVD.

Among the above-mentioned methods for CVD correction, glasses based on notch filters offer a non-invasive, immediate solution for enhancing color perception in individuals with red-green CVDs. These glasses work by selectively blocking narrow bands of overlapping wavelengths in the visible spectrum, thereby increasing the contrast between problematic color pairs such as red and green. Unlike electronic or medical interventions, notch filter-based glasses are passive optical devices that require no power or maintenance. Despite the commercial success and subjective appeal of notch-filter glasses, their clinical efficacy remains a subject of scientific debate. Several independent studies have evaluated the effectiveness of commercially available color-correction glasses based on spectral notch filtering. For example, Pattie et al. reported that EnChroma lenses produced noticeable changes in perceived color appearance but did not significantly improve performance on standard clinical tests such as the Ishihara plates or the D-15 panel test [21]. Similarly, Alvaro et al. demonstrated that although colored filters can modify the spectral composition of light reaching the retina, they cannot fully compensate for congenital color vision deficiency [28]. More recently, Somers et al. investigated multi-notch filters designed for deuteranomaly and reported that improvements in chromatic discrimination were highly variable across individuals [20]. These studies highlight that while spectral filters can enhance color contrast in some situations, their clinical benefits remain inconsistent and strongly dependent on the spectral characteristics of both the filters and the observer’s cone sensitivities.

The primary objective of this study was to quantitatively evaluate the clinical effectiveness of custom-fabricated notch filters in mitigating red-green CVD. By systematically varying the optical performance parameters, including center wavelengths, notch widths, and transmittance levels, we aimed to measure the resulting perceptual color discrimination changes in affected individuals. Through standardized tools such as the Farnsworth-Munsell (F-M) 100 Hue and D-15 panel tests, we established objective metrics, including total error score (TES), confusion angle, and confusion index (C-index), to distinguish between mere spectral changes and functional improvements in color ordering ability. Ultimately, this research seeks to identify how specific optical interventions translate into measurable clinical benefits, supporting the development of personalized color vision correction devices.

## 2. Materials and Methods

### 2.1. Participants

A total of 13 participants were recruited. Inclusion criteria included adults aged 20 to 65 years with either normal color vision or diagnosed CVD. Informed consent was obtained from all subjects. To ensure effective interaction with researchers during the test, participants were required to be able to fully communicate their feelings, reactions, and self-protection needs. Exclusion criteria included vulnerable populations as defined by the Fu Jen Catholic University Institutional Review Board (IRB), such as patients, pregnant women, minors, indigenous peoples, individuals with disabilities, prisoners, and individuals with high myopia (higher than −10.00 diopters). Participants with known ocular diseases that could affect visual quality or color perception, such as keratoconus, were also excluded. According to the IRB regulations of Fu Jen Catholic University, any research involving the measurement and recording of human sensory perception is classified as human subject research and requires ethical approval prior to initiation. All methods were performed in accordance with the relevant guidelines and regulations, as approved and verified by the Fu Jen Catholic University IRB. One participant with normal color vision was included only for the Color Bridge experiment to evaluate how the notch filters modify the perceived chromaticity of objects under controlled illumination conditions. This observer served as a reference group for characterizing the spectral and perceptual shifts introduced by the filters independent of color vision deficiency. This participant was not included in the clinical evaluation of color vision correction, such as the F-M 100 Hue or D-15 panel tests, which were conducted exclusively with participants diagnosed with CVD.

### 2.2. Clinical Trials

**Figure 1:**
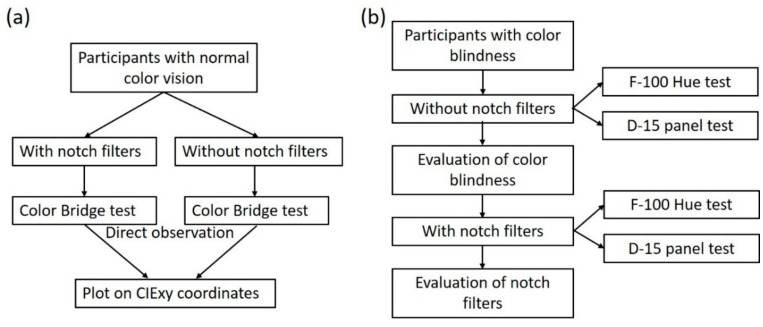
Flowcharts of (**a**) the Color Bridge tests, and (**b**) the panel tests for color vision.

**Figure 2:**
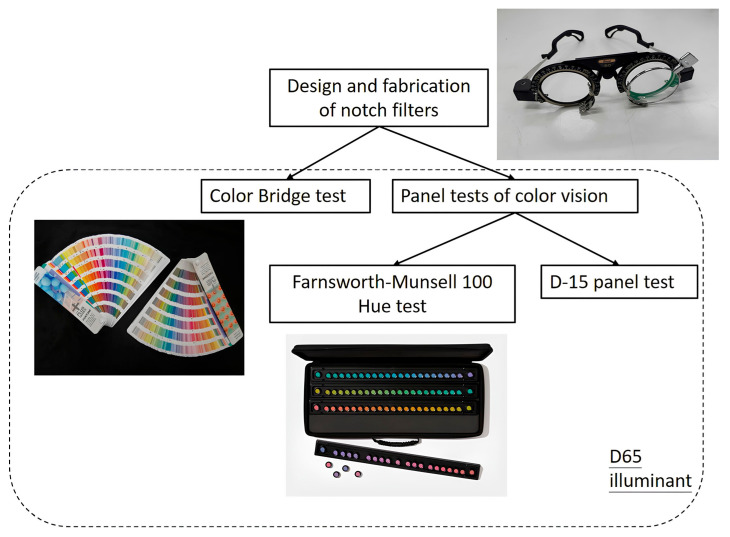
Notch filter glasses, color bridge test, and panel test of color vision used in the study.

This research was conducted as a prospective, quantitative clinical trial. The flowchart of this research is illustrated in Figure 1. Various notch filter glasses were fabricated and used as color blind glasses, as shown in Figure 2. These glasses were provided to participants, who wore them during the Color Bridge, F-M 100 Hue, and D-15 panel tests. All tests were conducted under a D65 standard illuminant. Participants were required to visually observe and distinguish colors and report their color perception through different methods. During testing, participants were monitored for any physical discomfort, such as dry or painful eyes. If such symptoms arose, participants were asked to rest with their eyes closed, or the experiment was immediately terminated.

**Figure 3:**
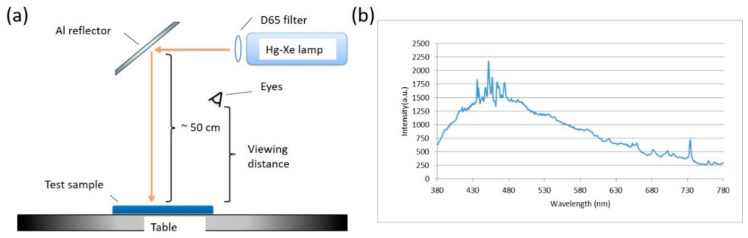
(**a**) Setup of the background light source. (**b**) Transmission spectrum of the light source after passing through the D65 filter. Intensity in a.u. refers to arbitrary units.

The setup of the background light source is illustrated in Figure 3a. A mercury-xenon lamp was used as the primary light source, and a D65 filter was applied to produce D65 background illumination. The corresponding spectrum is shown in Figure 3b. The light source was positioned approximately 50 cm above the table surface, and an aluminum reflector lined with crumpled aluminum foil was employed to diffuse the light uniformly across the table. Test samples were placed within the illuminated area, and participants observed them from a typical viewing distance. The measured average illuminance at this distance was approximately 235 lx.

### 2.3. Color Bridge Test

The Color Bridge test was designed to record and analyze the physical modification of reflected spectra independent of the neural anomalies present in color-deficient individuals. This experiment utilized the Pantone^®^ Color Bridge Guide Uncoated series (see Figure 2), which includes 1845 PANTONE spot colors on matte-coated offset paper.

#### 2.3.1. Experimental Design and Participants

To serve as a reference group for characterizing spectral and perceptual shifts, volunteers with verified normal color vision were recruited. Prior to the experiment, all participants underwent a baseline F-M 100 Hue test (see Section 2.5.1) to confirm the absence of CVD. The test was conducted using the following iterative matching procedure:

Filter Observation: A participant viewing through the 580 nm notch filter glasses (the “with filter” condition) observed six preselected color swatches.

Perceptual Matching: The participant without glasses (the “naked eye” condition) performed a matching task to identify six different swatches from the guide that most closely resembled the colors perceived by the participant wearing the filters.

Controlled Environment: As shown in Figure 3a, all observations were conducted under a D65 standard illuminant at a measured average illuminance of approximately 235 lx to ensure consistency.

#### 2.3.2. Data Analysis and Chromaticity Mapping

Following the matching process, the reflectance spectra of all swatches from both the “with filter” and “naked eye” conditions were measured. These results were projected onto the CIE 1931 chromaticity diagram, a two-dimensional map of human color perception that represents chromaticity coordinates (*x*, *y*) independently of luminance [29,30]. By calculating the angular difference in these coordinates relative to the white point, we quantified how the notch filter reshapes the spectral distribution and enhances the perceptual separation between colors on either side of the 580 nm reference line. This physical validation provided the necessary baseline for interpreting subsequent clinical evaluations in CVD participants.

### 2.4. Design of Notch Filters

Notch filters are designed to block light within a specific spectral band while maintaining high transmittance in adjacent wavelength regions. The key design parameters of a notch filter include the central wavelength, NW, and the transmittance level at the center of the rejection band [31,32]. A multilayer structure composed of two materials with different refractive indices, consisting of alternating high- and low-index layers with a quarter-wavelength optical thickness, respectively, can achieve high reflectance within specific wavelength bands. According to Epstein’s theory [32], the symmetric film stack can be equivalent to a single-layer film, which helps enhance transmittance outside the high-reflectance band and suppress unwanted spectral ripples.

Typically, the structure of the notch filter is based on a three-layer symmetric stack of the form ${\left(\frac{3H}{2}3L\frac{3H}{2}\right)}^{n}$ [33,34]. When expanded and excluding the first and last layers, the structure reveals a quarter-wavelength multilayer stack. *H* and *L* represent a high-refractive-index (*n_H_*) and low-refractive-index (*n_L_*) material with an optical thickness of *λ*_0_/4, respectively. *λ*_0_ is the center reference wavelength. The parameter *n* represents the number of symmetric periods. The full width at half maximum (FWHM) of the notch, 2*λ*_0_Δ*g*, referred to as the NW, can be determined from the refractive indices of the materials as [35], where

(1) $$△g=\frac{2}{\pi }{sin}^{-1}\left(\frac{n_{H}-n_{L}}{n_{H}+n_{L}}\right).$$

When *n* is large, the transmittance (*T_f_*) of *λ*_0_ is given by:

(2) $$T_{f}=\frac{16n_{0}n_{s}}{{\left(\frac{n_{H}}{n_{L}}\right)}^{2n}\left\{{{\left(n_{0}{+n}_{s}\right)}^{2}\left[\left(\frac{n_{0}n_{s}}{n_{H}}\right)-n_{H}\right]}^{2}\right\}}.$$

where *n*_0_ and *n_s_* denote the refractive indices of the ambient medium and the substrate, respectively, where *n_s_* = 1.5171, and *n*_0_ = 1. As shown in the above equations, the refractive indices of the materials determine the NW of the notch, whereas the number of layer periods *n* influences the transmittance at the center wavelength *λ*_0_.

In the study, the transmission spectra of the notch were designed with a narrow NW at *λ*_0_ from 550 to 590 nm. The film stack design is as ${\left(\frac{3H}{2}3L\frac{3H}{2}\right)}^{n}$, where *n* = 13, *H* and *L* represent Al_2_O_3_ and SiO_2_ layers with refractive indices of *n_H_* 1.6611 and *n_L_* 1.4587 at *λ*_0_ 580 nm, respectively. They were fabricated by an e-beam evaporator (SGC-22SA-IAD, SHOWA SHINKU, Sagamihara City, Japan). A B270 glass substrate with a diameter of 36 mm was used. The transmittance spectra of the as-deposited notch filters were measured using a Varian Cary 5E spectrophotometer in the wavelength range of 350–800 nm.

### 2.5. Panel Tests for Color Vision

To clearly assess the effects of color blind glasses on participants with CVD, two panel tests for color vision were employed, as illustrated in Figure 2: the F-M 100 Hue test and the D-15 panel test. Participants with clinically diagnosed CVD were recruited. Initially, they performed baseline measurements either unaided (naked eye) or with their own non-color-correcting glasses, using both tests. Test results were quantified using the TES (see Section 2.5.1) to indicate the severity of color confusion. The F-M 100 error distribution (see Section 2.5.1) and the D-15 panel confusion line diagrams (see Section 2.5.2) were used to evaluate the type and degree of CVD. Based on the identified confusion regions, suitable color blind glasses were selected for follow-up testing. After wearing the selected glasses, participants repeated both tests. Changes in TES values were then compared to assess the corrective effectiveness of the glasses. If no improvement was observed, alternative glasses were chosen based on the color vision evaluation, and retesting was conducted. If improvement was evident, testing was concluded, and the results were analyzed.

#### 2.5.1. Farnsworth-Munsell 100 Hue (F-M 100 Hue) Test

The F-M 100 Hue kit includes a set of 85 color caps distributed across four boxes, each containing 21 or 22 caps representing a continuous and uniform hue gradient (see Figure 1). The boxes correspond to hue transitions from red to orange, yellow to green, blue to purple, and purple to red. Each box is fitted with a black base to enhance color discrimination. At both ends of each box, two reference caps, representing the maximum hue difference, are fixed in position. Each cap is labeled with a number on the bottom to facilitate result recording. During the test, participants were asked to arrange the movable color caps between two fixed reference caps to form a continuous hue scale based on their visual perception. The final arrangement was used to evaluate the observer’s ability to discriminate hue differences. Owing to the nearly uniform color differences between adjacent caps, the F-M 100 Hue test provides evenly spaced data in the CIE chromaticity diagram [36]. Therefore, the test results could be quantitatively evaluated based on (1) the number of misplaced caps and (2) the positional error, defined as the distance between the actual and correct positions of each cap [36,37]. The TES is used to quantify performance by summing the individual cap error values, thereby eliminating the need for complex color difference calculations between adjacent caps. The TES values were calculated using the standard Farnsworth scoring method, in which the error score of each cap is determined based on the difference between adjacent caps in the final arrangement. The TES is computed as follows [38]:

(3) $$TES=\sum_{i=1}^{4}\left[\left(\sum_{j=1}^{n+2}{CE}_{j}\right)-2\left(n+2\right)\right],$$

(4) $$\mathrm{and}\;{CE}_{j}=\left|C_{j}-C_{j-1}\right|+\left|C_{j}-C_{j+1}\right|.$$

In these equations, *i* refers to the four boxes of caps (*i* = 1~4 for boxes A~D), *C_j_* is the numerical label of the *j*-th cap in the final sequence, CE*_j_* is the error value of the *j*-th cap, and *n* is the number of movable caps in each box. The two fixed reference caps at the ends of each box were excluded from the TES calculation because they are not movable caps. A lower TES value indicates a higher color discrimination ability. The severity of CVD based on TES was categorized according to established clinical norms: None to Slight (0–100), Moderate (101–180), and Severe (≥181) [36,37,38].

To minimize potential learning effects associated with repeated administration of the F-M 100 Hue test, participants did not perform practice trials prior to the baseline measurement. None of the participants reported prior experience with this test before enrollment in the study. The baseline test, therefore, represented the participants’ first exposure to the task. The second test was performed only after the participants wore the notch filter glasses, and the results were compared with the baseline measurements.

#### 2.5.2. D-15 Panel Test

The D-15 panel test is a simplified color arrangement task used to identify congenital CVD. It consists of 16 color caps: one fixed reference cap and 15 movable caps, each labeled with a unique number [39]. Participants were instructed to arrange the 15 caps in a hue gradient beginning with the reference cap. Upon completion, the arrangement was plotted on a CIE chromaticity diagram by sequentially connecting the coordinates of each cap [40]. The number and orientation of the resulting line segments, particularly those aligned with confusion lines (loci), indicate the type and severity of the participant’s CVD [40]. The confusion lines refer to specific directions in color space along which individuals with CVDs confuse hues. These lines represent the axes of perceptual error caused by deficiencies in the cone photoreceptors (L, M, or S cones). Each type of CVD, protan (red), deutan (green), or tritan (blue), has its own confusion line, corresponding to the red-green or blue-yellow axes in a chromaticity diagram. When arranging the D-15 test caps, individuals with CVD tend to make characteristic swaps along these lines. By analyzing the pattern and angle of these errors, clinicians can identify the type and severity of CVD. In this study, both physical and online versions of the D-15 panel were utilized. The physical version was a custom-designed set consisting of 16 caps selected from the F-M 100 Hue set. The online version was administered on a laptop using the Color Arrangement Test from the Colblindor website (see https://www.color-blindness.com).

### 2.6. Mathematical Simulation of CVD for Notch Filter Design

**Figure 4:**
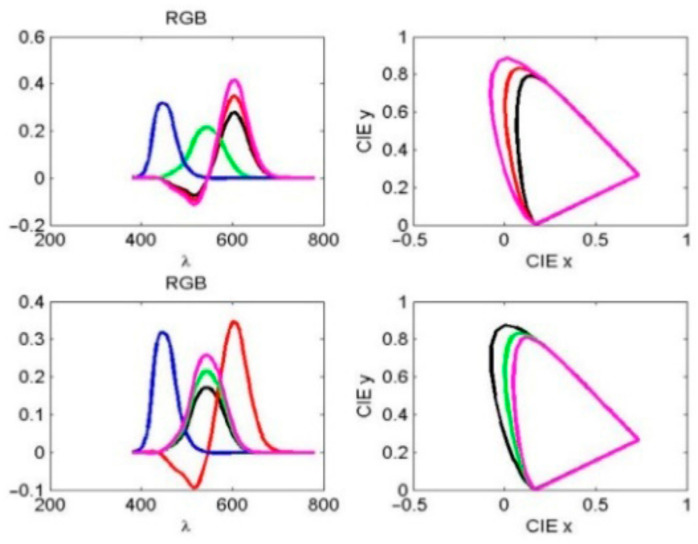
(**Left**): Observed spectra of red, green, and blue light in the RGB color space. (**Right**): The corresponding CIE xy plots. The black and magenta curves indicate that the amplitude varies by 0.8 and 1.2 times of the normal red (**top**) and green (**bottom**) curves, respectively.

The observed spectra of red (R), green (G), and blue (B) light in the RGB color space correspond to wavelength ranges of 560–580 nm, 530–540 nm, and 420–440 nm, respectively, as shown in Figure 4. Simulated stimulation of cone cells in response to R and G light was modeled by varying the normal red and green response curves by 0.8 and 1.2 times, respectively, as indicated by the black and magenta curves in the same figure.

The remaining XYZ tristimulus values in the CIE 1931 color space (CIE-XYZ coordinates), as defined by the International Commission on Illumination (CIE), are nonnegative and are obtained by transforming the spectral power distribution I(λ) according to [41]

(5) $$\left\lfloor \begin{array}{c} X\\ Y\\ Z \end{array}\right\rfloor =\frac{1}{0.17697}\left[\begin{array}{ccc} 0.49 & 0.31 & 0.2\\ 0.17697 & 0.8124 & 0.01063\\ 0 & 0.01 & 0.99 \end{array}\right]\left\lfloor \begin{array}{c} R\\ G\\ B \end{array}\right\rfloor .$$

**Figure 5:**
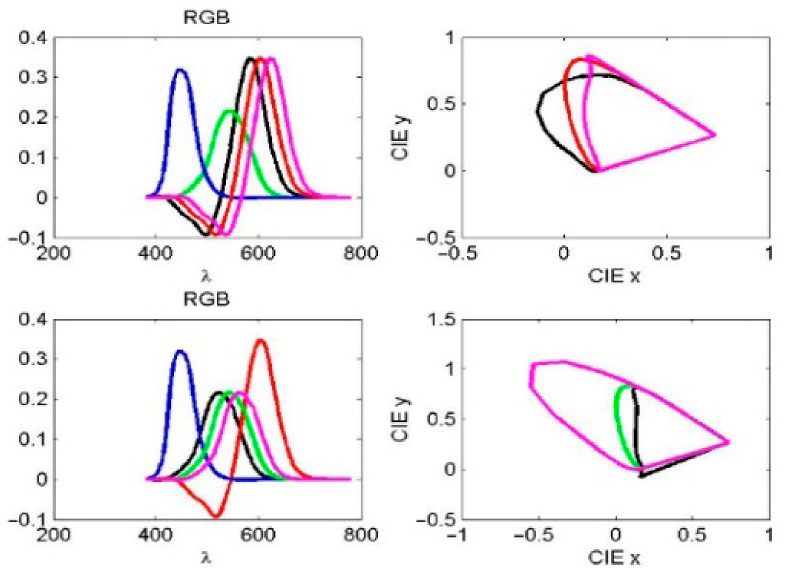
(**Left**): Observed spectra of red, green, and blue light in the RGB color space. (**Right**): The corresponding CIE xy plots. The black and magenta curves represent the left and right 20 nm deviations of the normal red (**top**) and green (**bottom**) curve spectra, respectively.

The normalized color space CIE-xyz is read as x = X/(X + Y + Z), y = Y/(X + Y + Z), z = Z/(X + Y + Z). The effects of notch filter amplitude and spectral shift on the R and G light curves were analyzed using CIE xy color space representations. For individual CVDs, the black and magenta curves in both the RGB and CIE xy plots in Figure 4 represent amplitudes of 0.8 and 1.2 times the normal red and green curves, respectively. As illustrated in Figure 5 left, the black and magenta curves correspond to 20 nm leftward and rightward spectral shifts in the normal red and green spectra, respectively. These two simulations illustrate the unnormalized R and G responses, the resulting boundary curves, and the corresponding color coordinates in the CIE xy color space (see Figure 4 right and 5 right).

## 3. Results

### 3.1. Characterization of Notch Filters

**Figure 6:**
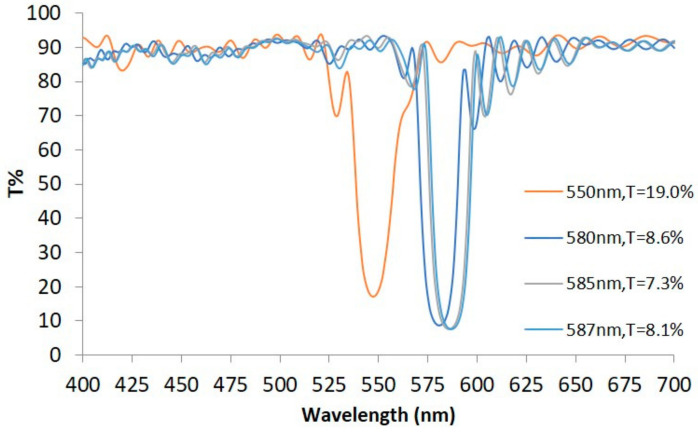
Transmission spectra of notch filters with four different central wavelengths.

Since congenital CVDs are primarily caused by anomalies in the L and M cone cells, the overlapping spectral region of these two cones, approximately 550 nm to 590 nm, was selected as the central wavelength range for fabricating several notch filters. Using optical monitoring, notch filters were deposited with four different central wavelengths: 550 nm, 580 nm, 585 nm, and 587 nm. The center transmittance values, from left to right in Figure 6, are approximately 19.0%, 8.6%, 7.3%, and 8.1%, whose numbers of symmetric periods (*n*) are 9, 13, 13, and 13, respectively, with approximately 20 nm NWs. The transmittance generally ranges between 70% and 90% outside the notch regions. Since the transmittance *T_s_* of the other surface of the substrate is not 1, but 0.958, the transmittance value of the notch filter is lower than the designed *T_f_* of Equation (2).

### 3.2. Results of the Color Bridge Tests

As described in Section 2.6, the CIE xy color space can be altered by modifying the relative responses of the R and G channels. To verify this change, a 27-year-old male participant with normal color vision underwent testing using a 580 nm notch filter. The identification numbers and corresponding RGB values of the preselected color swatches observed by the participant, both with and without the filter, are listed in Table 1.

**Figure 7:**
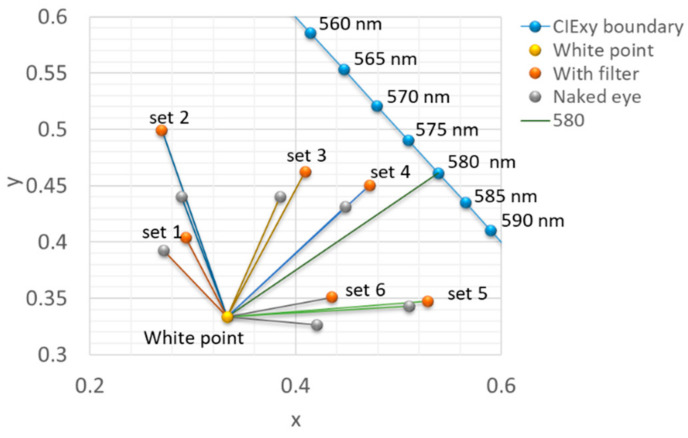
CIE xy diagram for the Color Bridge test illustrating the physical modification of perceived chromaticity coordinates using the 580 nm notch filter. Gray circles represent the baseline coordinates of Pantone swatches perceived by the naked eye, while red circles represent the coordinates of matched swatches viewed through the filter. Lines connecting these points to the central white point indicate the direction and magnitude of the perceptual shift. The 580 nm line serves as a reference.

The RGB values were first converted into the 3D CIE-XYZ color space and then projected onto the 2D CIE xy space, as shown in Figure 7 [42,43]. Coordinates for the with (red circles)/without filter (gray circles) were calculated and connected to the white point by solid lines. The theoretical XYZ tristimulus values of all color swatches were calculated as follows:

(6) $$\{\begin{array}{c} X=k\int_{380}^{780}S\left(\lambda \right)R(\lambda )T(\lambda )\bar{x}(\lambda )d\lambda \\ Y=k\int_{380}^{780}S\left(\lambda \right)R(\lambda )T(\lambda )\bar{y}(\lambda )d\lambda \\ Z=k\int_{380}^{780}S\left(\lambda \right)R(\lambda )T(\lambda )\bar{z}(\lambda )d\lambda \end{array},$$

where k is a normalization constant, S(λ) is the spectrum of the light source, R(λ) is the reflected spectrum of the color swatch, T(λ) is the transmittance spectrum of the 580 nm filter, and $\bar{x}\left(\lambda \right),\bar{y}\left(\lambda \right),and\bar{z}(\lambda )$ are the color matching functions [42,44]. After being converted to the 2D CIE xy space, the theoretical coordinates are represented as dark blue circles in Figure 7.

For six sets of color swatches, the experimental coordinates in two groups are listed in Table 1. The variation from the “without filter” (gray circle) and “with filter” (red circle) is represented by the angular difference. The positive value indicates a clockwise offset corresponding to the “White point”. After using a 580 nm notch filter, the angle changes in Sets 1–4 shift clockwise, and the angle changes in Sets 5–6 shift counterclockwise, with 580 nm as the reference line. This suggests that the 580 nm filter enhances the color difference between colors on either side of the reference line. Furthermore, the angles closer to the white point (e.g., Sets 1 and 6, which have relatively high light intensity) are larger than the angles farther away from the white point (e.g., Sets 2 and 5, which have relatively low light intensity). From this experiment, the color discrimination ability of the CVD could be improved by the various notch filters.

This test was conducted with a single participant possessing normal color vision to serve as a reference group for characterizing the spectral and perceptual shifts introduced by the filters. The objective of this specific experiment was to characterize the physical modification of reflected spectra independent of the neural and physiological anomalies present in color-deficient individuals. By recording how the 580 nm filter alters perceived chromaticity coordinates for a standard observer, we were able to verify the theoretical model of spectral sharpening and confirm that the filters enhance angular separation in the CIE xy chromaticity diagram. This physical validation provided the baseline necessary for interpreting the subsequent clinical evaluations (F-M 100 Hue and D-15 tests), which were conducted exclusively with a larger cohort of participants diagnosed with various phenotypes of CVD.

### 3.3. Results of the F-M 100 Hue Tests

This phase of testing recruited 12 male subjects aged 20 to 65 years. Baseline color vision status was evaluated using the F-M 100 Hue test prior to the intervention. According to the TES, participants were classified as having normal color vision to slight CVD, moderate, or severe CVD. The baseline characteristics of the participants are summarized in Table 2.

Four different notch filters (550, 580, 585, and 587 nm) were used. The light intensity could decrease after passing through multiple notch filters (e.g., two 580 nm notch filters: 580 × 2, 550-580-585, and 580 × 2-585). The test results, for both naked eye and various filter combinations, were entered into the software provided with the test kit, which generated the TES and error distribution maps shown in Table 3. The red words indicate an increased TES (worsening) after wearing the correction glasses. The degree of improvement was calculated as the percentage difference between the two TES values. The results showed 10 cases of improvement from 6.67% to 50.00%, 2 cases of worsening (−4.4% and −16%). These findings indicate that the notch filters improved color discrimination for most subjects, although the degree of improvement varied individually and showed no clear correlation with the original severity of CVD.

Table 3 clearly indicates patterns of color confusion. Some cases showed clear improvement or deterioration before and after wearing the glasses. For a more detailed analysis of the error distribution maps, each map was divided into the left half in the red/yellow/green region and the right half in the blue-green/blue/purple region. These regions roughly correspond to the red/yellow/green and blue-green/blue/purple color ranges, respectively. It was also noted that error distribution maps from different participants viewing with the naked eye exhibited similar patterns. In Table 3, error distribution maps (with the naked eye) were categorized into the three types described above.

(1) Uniform distribution type such as No. 1, 2, 6, and 10. Errors were evenly distributed, with low scores mostly below 5 and no distinct peaks. The average improvements of the 580 nm filter exceeded 35.7%, except for No. 10.

(2) Right-hemisphere concentrated type, such as No. 5, 7, and 11. Larger errors predominantly appeared in the right half of the map, with concentrated areas varying among subjects. The left half of the map also showed errors, but with less pronounced magnitudes than the right half. The improvements of the 580 nm filter exhibited worsening in two cases. The improvements of the 580 and 587 nm filters were greater than 18%.

(3) Both-Hemisphere Concentrated Type, such as No. 3, 4, 8, 9, and 12. Significant errors appeared in both left and right halves, with concentrated areas varying by individual. This type had two improvements, two worsens, and one unchanged case. The average improvements of the 580 and 587 nm filters were about 14%, while two worsened cases of No. 4 and 9.

The 580 nm filter performed well in the left hemisphere, with 7 cases showing improvement and 4 showing deterioration. Specifically, there were 5 improvements, 2 unchanged cases, and 4 deteriorations in the right half. Other filter combinations resulted in 3 improvements and 1 deterioration in the left half, and 1 improvement and 3 deteriorations in the right half.

In summary, the notch 580 nm filters were more effective in improving performance in the red/yellow/green region. The greatest effect for Type (1) and the least in the blue-green/blue/purple region. TES changes quickly indicate whether the notch filters produce improvement, but TES alone cannot specify which colors the subjects confuse.

### 3.4. Results of the D-15 Panel Tests

**Figure 8:**
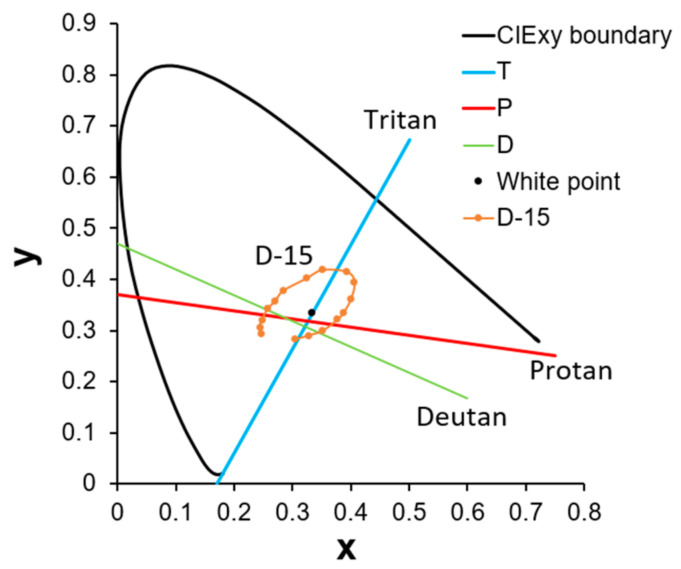
Confusion lines for deutan (D), protan (P), and tritan (T) in CIE xy coordinate. The lines D (deutan), P (protan), and T (tritan) represent the axes of perceptual error caused by specific cone photoreceptor deficiencies. The brown sequence (D-15) illustrates a standard circular arrangement for a normal observer. In individuals with CVD, arrangement sequences typically collapse into segments parallel to these axes, allowing for the quantitative classification of the participant’s deficiency type.

Among the 12 participants who took the F-M 100 Hue test, 6 volunteers (Subjects 4, 5, 6, 8, 9, and 12) were selected to participate in the D-15 panel test. The confusion line diagrams were generated based on each subject’s color arrangement order. These diagrams were subsequently analyzed using vector analysis to produce vector confusion diagrams, from which the confusion angle, TES, C-index, and other indicators were calculated. Finally, these data were used to evaluate the corrective effect of the 580 nm filter. Before plotting the confusion line diagram, the confusion lines corresponding to various types of CVDs must first be defined. As shown in Figure 8, three confusion lines closest to the white point and encompassing the largest areas in both the online and physical coordinate sets were selected as reference lines. Specifically, lines D, P, and T represent the deutan (green deficiency), protan (red deficiency), and tritan (blue deficiency) confusion lines, respectively. The color caps were then connected according to the subjects’ arrangement order on the CIE xy coordinate system. Since the online and physical tests yielded similar results, only the findings from the physical version are reported.

The results are shown in Table 4. Line segments in the diagram that are approximately parallel to specific confusion lines were identified. A greater number of parallel segments associated with a particular type of CVD indicates a more pronounced presence of that deficiency in the participant. Classification results are presented in Table 4, with ambiguous cases marked by two closely related categories. In this table, “N” indicates normal or slight misplacement with low error rates and is thus classified as normal, while “D” and “P” represent deutan and protan types, respectively. As indicated, some cases exhibited weakened original CVD characteristics or even approached normality after correction with the notch filter. For example, participants 5 and 12 showed a marked reduction in lines parallel to the green confusion line after correction, indicating reduced deutan traits. Participant 9 displayed alleviated protan symptoms. However, participant 6 exhibited two adjacent cap misplacements with the 580 nm filter, resulting in slightly lower accuracy than the naked-eye measurement. Other cases showed no obvious features, making the correction effect difficult to assess.

To obtain clearer metrics for analysis, a scoring method was applied to the D-15 panel test by calculating the moment of inertia from the confusion line diagram, as detailed in the reference [40]. Using the CIELUV coordinate system, each connection between color caps in the confusion diagram was represented as a vector, with the origins of all 15 vectors translated to the coordinate origin. Principal (major) and secondary (minor) radii were then calculated by treating all vectors as a rigid body with unit mass concentrated at their endpoints. Using these two radii, the following indicators were defined and calculated [40]:

(1) Confusion Angle is the angle of the principal radius, used to estimate the subject’s type of CVD. An angle threshold of 0.7° is used to distinguish between protan (>0.7°) and deutan (<0.7°) types. Tritan is indicated by angles less than −65°.

(2) Scatter Index (S-index) is the ratio of the principal to secondary radii and describes the dispersion of color vectors. A higher value indicates stronger alignment of vectors along the principal radius, implying more distinct color confusion characteristics.

(3) Confusion Index (C-index) is the ratio of the subject’s principal radius length to that of the correctly arranged D-15 panel, quantifying the severity of CVD. Values between 1.0 and 1.6 are considered normal or mild; values greater than 1.6 indicate congenital color vision deficiency.

Table 5 presents these indicators for 6 participants before and after wearing the 580 nm filter. Values corresponding to normal (correct) D-15 arrangements are shown in blue font for reference. Participants were categorized into four types based on these indices: no deficiency (normal), deutan, protan, and tritan. Improvements, indicated by a decrease in the C-index, are highlighted with green boxes; deteriorations, indicated by an increase in the C-index, are shown in red boxes. To determine the type of CVD, the C-index is first considered: values below 1.6 indicate correct or nearly correct color ordering and are classified as normal. Values above 1.6 indicate a deficiency and are further classified based on the confusion angle. Across the table, four cases showed improvement, and two showed deterioration after wearing the glasses. Participant 5 shifted from deficient to normal vision. Two participants maintained their deficiency type but showed a reduced C-index, and two changed from deutan to protan.

**Figure 9:**
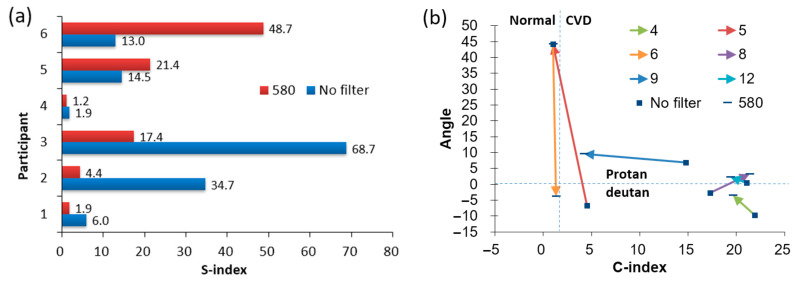
(**a**) S-index values, and (**b**) C-index values versus confusion angles for 6 participants before and after wearing the 580 nm filter. Part (**a**) compares the S-index, which measures the dispersion of color vectors; a lower value indicates that vector angles are more evenly distributed, reducing the tendency toward a specific color confusion. Part (**b**) tracks individual trajectories of clinical improvement. The vertical dashed line (C-index = 1.6) separates the normal (left) and color-deficient (right) regions, while the horizontal dashed line (0.7°) distinguishes protan (above) from deutan (below) types. Leftward-moving arrows (e.g., Participant 5) indicate improved color discrimination, with some participants shifting from the deficient region into the normal region while wearing the 580 nm filter.

The S-index measures the dispersion of color vectors; higher values indicate lower vector consistency. Figure 9a shows the S-index values for participants under both naked-eye and 580 nm filter conditions. The results indicate that 4 out of 6 subjects exhibited decreased S-index values when wearing the glasses. This suggests that the 580 nm filter generally causes the vector angles to be more evenly dispersed in all directions, rather than clustering around a single confusion angle, thereby reducing the subject’s tendency toward a specific color deficiency. Figure 9b shows the changes in confusion angle and C-index before and after wearing the 580 nm filter [45]. Solid colored lines represent each subject’s changes; squares indicate naked-eye values, while short horizontal bars represent values with the glasses. The vertical dashed line at C-index = 1.6 separates the normal (left) and color-deficient (right) regions. The horizontal dashed line at confusion angle = 0.7° distinguishes protan (above) from deutan (−65° to 0.7°) types. Among the plotted trajectories, four cases shifted leftward, indicating improved color ordering ability. One of these crossed the C-index 1.6 boundary from the deficient to the normal region, demonstrating the 580 nm filter’s effectiveness in improving color discrimination. Vertical movements reflect changes in the confusion angle, with five cases moving upward and one downward. Two cases crossed the 0.7° threshold, changing classification from deutan to protan, indicating that the 580 nm filter tends to shift confusion angles toward the protan type.

## 4. Discussion

Compared with previous evaluations of commercially available color-correction glasses, the present study focuses on the relationship between filter design parameters and quantitative clinical outcomes. Many commercial products employ proprietary multi-notch spectral profiles whose optical parameters are not publicly disclosed, making systematic analysis difficult. In contrast, the filters used in this study were fabricated with controlled center wavelengths and bandwidths using multilayer thin-film deposition. This approach allows direct investigation of how specific spectral filtering parameters influence perceptual color discrimination. By combining chromaticity analysis in CIE color space with standardized clinical tests (F-M 100 Hue and D-15), the present work provides a framework for linking optical filter design to measurable changes in color discrimination performance.

The efficacy of notch filter-based glasses in correcting red-green CVD is based on the principle of spectral sharpening. In individuals with anomalous trichromacy, the spectral sensitivities of the long-wavelength (L) and medium-wavelength (M) cones are abnormally close, leading to substantial overlap in the 530–590 nm range. This overlap causes the brain to receive nearly identical signals for different wavelengths, forming the physiological basis of color confusion. The notch filters were designed to target this region of overlap, creating a narrow-band spectral “gap” that selectively reduces simultaneous stimulation of both L and M cones. By diminishing neural crosstalk between these cone channels, the filters amplify the relative difference in response intensity for colors on either side of the notch. This enhanced chromatic contrast increases perceptual separation along the confusion lines in color space, which the visual cortex interprets as improved color discrimination. The optical mechanism underlying this effect explains the observed shifts in chromaticity coordinates and the enhanced angular separation in the CIE xy diagram during the Color Bridge test, confirming the filters’ effectiveness in improving color perception in individuals with red-green CVD.

The results from the Color Bridge experiment demonstrated that the 580 nm notch filter altered the perceived chromaticity of selected color swatches, producing measurable angular differences relative to the white point in the CIE xy chromaticity diagram (see Figure 7). These findings indicate that spectral filtering can modify the effective color stimulus reaching the retina and thereby alter the perceived color relationships among objects. Such effects are consistent with theoretical predictions that notch filters can reshape the spectral distribution of light and amplify perceptual differences along color confusion axes.

Clinical evaluation using the F-M 100 Hue test further demonstrated that most participants experienced improvements in color discrimination when wearing the correction glasses (see Table 3). The F-M 100 Hue test was used primarily to quantify color discrimination performance rather than to establish a clinical diagnosis of CVD. Approximately 83% of subjects exhibited reduced TES, with improvements ranging from 6.67% to 50.00%. These results are broadly consistent with previous studies reporting that spectral filtering lenses can enhance color discrimination performance in individuals with anomalous trichromacy by increasing chromatic contrast between confusing color pairs. However, the degree of improvement varied among participants, reflecting the heterogeneity of CVD conditions and the differing spectral characteristics of individual cone photoreceptor anomalies. In addition, two subjects (No. 4 and 9) exhibited a deterioration in their TES. This lack of uniform success is likely due to the spectral heterogeneity of anomalous trichromacy. Corrective efficacy depends on the alignment between the filter’s narrow-band ‘gap’ and the specific wavelength overlap of the user’s L and M cone sensitivities. If a participant’s spectral sensitivity peak is significantly shifted away from the 580 nm design, the filter may fail to sufficiently reduce neural crosstalk. Furthermore, the deterioration in performance for specific subjects may be attributed to other factors: (1) a reduction in overall retinal luminance, which is inherent to passive optical filters and can impair hue discrimination in certain phenotypes, and (2) shifts in the confusion angle. As observed in the D-15 analysis, the 580 nm filter tends to shift confusion angles toward the protan type. For some individuals, this redistribution of chromatic information may introduce new perceptual confusions that outweigh the benefits of enhanced contrast. These findings underscore that notch filter glasses primarily act by increasing chromatic contrast rather than restoring normal trichromatic vision, highlighting the necessity for personalized spectral optimization based on individual diagnostic data.

The D-15 panel test provided additional insight into how notch filters influence color confusion patterns. Vector analysis of the confusion diagrams showed that several participants exhibited decreased C-index and S-index when wearing the 580 nm filter. In one case, the C-index decreased to a value corresponding to normal color discrimination. These results suggest that the notch filters can partially reduce the strength of confusion lines by redistributing chromatic information across the color space. Nevertheless, some cases showed little improvement or even slight deterioration, indicating that spectral filtering may shift the direction of color confusion rather than fully correcting the underlying photoreceptor deficiency. This observation is consistent with previous reports that color correction glasses primarily enhance contrast rather than restore normal trichromatic vision. In addition, Subjects 8 and 12 showed a shift from deutan to protan. It is critical to clarify that such a shift does not imply a biological “cure” or a transformation of the underlying genetic deficiency. Rather, the observed transition from deutan to protan classification is a byproduct of how the notch filter artificially reshapes the visible spectrum. By selectively blocking the 550–590 nm range, the filter attenuates the specific neural signals that lead to green-deficiency (deutan) patterns, but this inevitably leaves the observer with a “skewed” spectral input that may then mimic the characteristics of red-deficiency (protan).

In this study, participants were not classified using pseudoisochromatic plate tests such as Ishihara or anomaloscope-based Rayleigh matching. While the F-M 100 Hue and D-15 tests provide quantitative measures of color discrimination, they are not considered definitive diagnostic tools for determining the precise subtype of red-green color vision deficiency. Future studies will incorporate standard diagnostic tests such as Ishihara plates and anomaloscope measurements to more accurately classify participants.

Compared with previously reported color vision correction approaches, the notch filter strategy offers several practical advantages. Unlike electronic assistive devices or gene therapy approaches, optical filters provide an immediate, non-invasive method for enhancing color perception. Previous investigations of multi-band notch filter glasses, such as those used in commercial products, have demonstrated varying degrees of effectiveness depending on lighting conditions and the specific type of color vision deficiency. The present results support these findings and provide additional quantitative evidence using standardized clinical tests and chromaticity analysis.

Despite these encouraging findings, several limitations of this study should be acknowledged. The relatively small number of participants limits the statistical power and generalizability of the results. In addition, the cohort included individuals with different types and severities of CVD, which may have contributed to the variability in responses to the notch filters. The evaluation was also primarily conducted using laboratory-based color discrimination tests under controlled illumination conditions; therefore, the performance of the filters in real-world visual tasks, such as object recognition or navigation in natural environments, was not assessed. Furthermore, the selection of the most suitable notch filter for each participant was based on observed changes in color discrimination metrics during testing. Although this approach allowed exploration of individualized spectral optimization, it may introduce potential selection bias. Another limitation is that the analysis relied mainly on descriptive comparisons of test results obtained before and after the use of the notch filters. Because of the limited sample size and the substantial inter-individual variability in responses among participants with different CVD types and severities, formal group-level statistical testing was not performed. Consequently, the present findings should be interpreted as preliminary and exploratory. Future studies should address these limitations by recruiting larger participant cohorts and performing more detailed classifications of CVD using genetic or physiological diagnostic methods. Systematic optimization of the spectral parameters of the notch filters, including center wavelength, bandwidth, and transmittance, may further enhance their effectiveness for specific CVD subtypes. In addition, integrating spectral modeling with larger datasets of patient responses may enable the development of personalized color-correction lenses tailored to individual visual characteristics, thereby advancing precision optical solutions for color vision deficiency.

Another important consideration is the ongoing scientific debate regarding the effectiveness of color-correcting glasses. Several studies have suggested that while spectral filters can alter perceived colors, they do not necessarily restore normal color discrimination in individuals with congenital color vision deficiency. For example, investigations of commercial multi-notch filters have reported that users often experience noticeable changes in color appearance or subjective color vividness, yet improvements in standardized clinical tests such as the Ishihara plates or the D-15 panel test are often limited or inconsistent [21,28]. These findings have led some researchers to argue that such filters primarily modify the spectral composition of the light reaching the retina, thereby shifting perceived chromatic relationships rather than correcting the underlying cone photoreceptor deficiency. Consequently, the observed benefits may arise from enhanced chromatic contrast between certain color pairs rather than a true restoration of trichromatic color vision. The results of the present study are broadly consistent with this interpretation: although many participants showed improved performance in quantitative color discrimination tests, the magnitude of improvement varied among individuals, and in some cases, the confusion patterns were shifted rather than completely eliminated. These observations highlight that notch filter glasses should be regarded as perceptual aids that can enhance color contrast for some users rather than as devices that fully correct the physiological basis of CVD.

## 5. Conclusions

In this study, multilayer notch filters were successfully designed and fabricated for use in color vision correction glasses. Clinical evaluations using Color Bridge and panel tests demonstrated that these filters can alter color perception and enhance chromatic discrimination to varying degrees. Specifically, the 580 nm filter improved color discrimination performance by reducing both C-index and S-index values in most subjects. However, the researchers emphasize that these filters act as perceptual aids that increase chromatic contrast rather than devices that fully correct the underlying physiological basis of color vision deficiency. Results varied among individuals depending on the type and severity of their condition, often resulting in a shift in confusion patterns toward protanomaly rather than a total restoration of normal trichromatic vision. These preliminary findings suggest that while notch filters provide a non-invasive method for enhancing color perception, future development will require personalized spectral optimization and larger data sets to tailor eyewear to individual visual characteristics. Rather than demonstrating superiority over existing commercial products, this study aims to provide quantitative insight into how specific spectral filtering parameters influence clinical measures of color discrimination.

## Figures and Tables

**Table 1:** Comparing color swatches observed by a normal participant without/with the 580 nm filter.

	Naked Eye				With Notch Filter				
Set	R	G	B	CIE x, y	R	G	B	CIE x, y	Angle Diff. (°)
1	71	215	172	0.29, 0.44	128	224	167	0.27, 0.50	16.45
2	0	150	57	0.27, 0.39	0	177	64	0.29, 0.40	1.59
3	226	232	104	0.38, 0.44	236	232	26	0.41, 0.46	4.87
4	241	180	52	0.45, 0.43	255	198	0	0.47, 0.45	0.33
5	192	13	30	0.51, 0.34	230	52	34	0.53, 0.35	−1.07
6	255	128	139	0.42, 0.33	255	134	116	0.43, 0.35	−14.36

**Table 2:** Baseline characteristics of the study participants.

Characteristic	Value
Participants included in color vision panel tests	12
Sex	Male: 12
Age (years)	20–65
Color vision status (baseline F-M 100 Hue test)	
Normal to Slight CVD	4
Moderate CVD	7
Severe CVD	1
Type of color vision deficiency (D-15 panel test subset, n = 6)	
Deutan type	3
Protan type	1
Mixed/uncertain	1
Normal	1

**Table 3:** TES results from the F-M 100 Hue test for 12 participants before and after wearing the best correction glasses.

No.	1	2	3	4	5	6
TES, naked	24	92	92	92	120	140
CVD severity	N-S	N-S	N-S	N-S	M	M
Error distribution maps (naked)	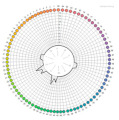	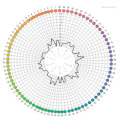	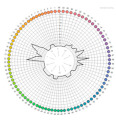	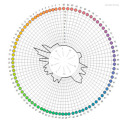	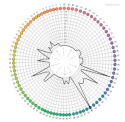	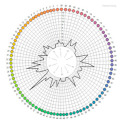
Notch filter	580	580	580	587	587	580
Error distribution maps (filter)	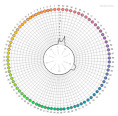	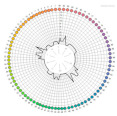	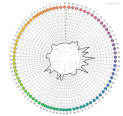	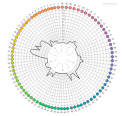	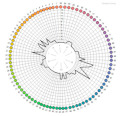	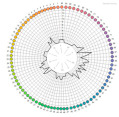
TES, filter (Improvement)	12 (50%)	68 (26%)	68 (26%)	96 (−4.4%)	96 (20%)	96 (31%)
**No.**	**7**	**8**	**9**	**10**	**11**	**12**
TES, naked	144	148	152	168	172	188
CVD severity	M	M	M	M	M	Se
Error distribution maps (naked)	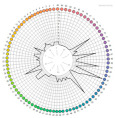	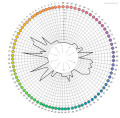	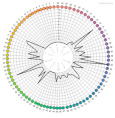	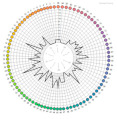	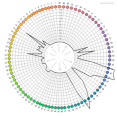	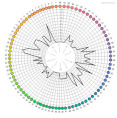
Notch filter	580	580	580	580 × 2	587	580
Error distribution maps (filter)	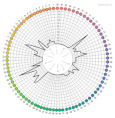	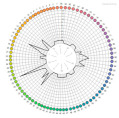	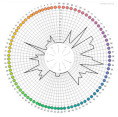	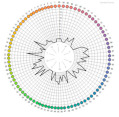	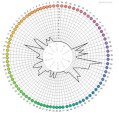	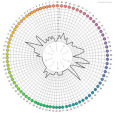
TES, filter (Improvement)	116 (19%)	96 (34%)	176 (−16%)	152 (9.5%)	144 (16%)	128 (32%)

(for CVD severity, N-S: None to Slight; M: Moderate; Se: Severe).

**Table 4:** Confusion line diagrams generated from the physical D-15 panel test results.

No.	4	5	6	8	9	12
Naked Confusion type	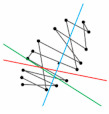 D	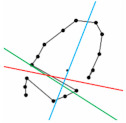 D	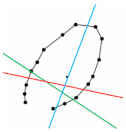 N	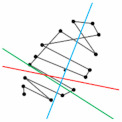 D	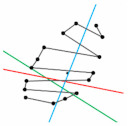 P	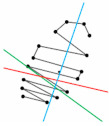 DP
580 Filter Confusion type	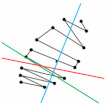 D	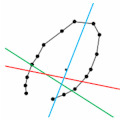 N	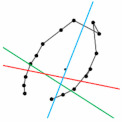 N	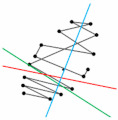 DP	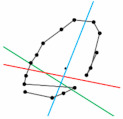 NP	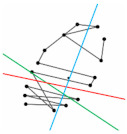 P

**Table 5:** Confusion angles, S-indices, and C-indices for 6 participants before and after wearing the 580 nm filter.

No.		Angle	Principal Radius	Secondary Radius	S-Index	C-Index	CVD Type
	Normal	44.2	11.1	6.0	1.86	1.00	no
4	Naked	−9.7	42.8	3.3	13.04	21.90	deutan
	580	−3.5	37.9	0.8	48.67	19.66	deutan
5	Naked	−6.7	16.6	2.8	5.97	4.55	deutan
	580	44.2	11.1	6.0	1.86	1.00	no
6	Naked	44.2	11.1	6.0	1.86	1.00	no
	580	−3.8	13.2	11.0	1.20	1.34	no
8	Naked	−2.6	35.8	2.5	14.55	17.29	deutan
	580	3.3	40.8	1.9	21.41	21.36	protan
9	Naked	6.9	28.9	0.8	34.66	14.76	protan
	580	9.7	17.0	3.9	4.38	4.21	protan
12	Naked	0.4	38.4	0.6	68.74	21.06	deutan
	580	2.2	38.3	2.2	17.42	19.36	protan

## Data Availability

Data will be made available upon reasonable request to the corresponding authors.
